# Supplementary material for: P-TRAP: a Panicle Trait Phenotyping tool
Source: BMC Plant Biol. 2013 Aug 29;13:122. doi: 10.1186/1471-2229-13-122 (PMC3848748; doi:10.1186/1471-2229-13-122)
Supplement: Additional file 7 — P-TRAP source code. The Netbeans project that contains the source code of the application. [file 1471-2229-13-122-S7.zip › javadoc.zip!/javadoc/org-basmati-workspace/org/basmati/workspace/GrainsFinder.html]

GrainsFinder (WorkSpace)


JavaScript is disabled on your browser.


- Package
- Class
- Tree
- Deprecated
- Index
- Help

*org.basmati.workspace 1.0*

- Prev Class
- Next Class

- Frames
- No Frames

- All Classes

- Summary:
- Nested |
- Field |
- Constr |
- Method

- Detail:
- Field |
- Constr |
- Method


org.basmati.workspace

## Class GrainsFinder

- java.lang.Object
- - org.basmati.workspace.GrainsFinder

- ---

    

  ```
  public abstract class GrainsFinder
  extends java.lang.Object
  ```

  Grains finder finds the grains in both a single image and a set of images. It
  is very similar to the GrainTraitsFinder class but they use different methods
  and the latter is faster is designed for uniform distributed grains.

  Author:
  :   faroq

  See Also:
  :   `GrainTraitsFinder`

- - ### Constructor Summary

    Constructors

    Constructor and Description

    `GrainsFinder()`
  - ### Method Summary

    Methods

    | Modifier and Type | Method and Description |
    | --- | --- |
    | `static void` | `findgrains(org.openide.filesystems.FileObject fileObject)` Detect the grains in the branches. |
    | `static void` | `findgrains(org.openide.filesystems.FileObject[] fileObjects)` Detect the grains of a set images. |

    - ### Methods inherited from class java.lang.Object

      `clone, equals, finalize, getClass, hashCode, notify, notifyAll, toString, wait, wait, wait`

- - ### Constructor Detail


    - #### GrainsFinder

      ```
      public GrainsFinder()
      ```
  - ### Method Detail


    - #### findgrains

      ```
      public static void findgrains(org.openide.filesystems.FileObject fileObject)
      ```

      Detect the grains in the branches. This is a single image detector
      version.

      Parameters:
      :   `fileObject` -

      See Also:
      :   `GrainTraitsFinder`,
          `GrainTraitsFinder.findGrainsTraits(org.openide.filesystems.FileObject)`


    - #### findgrains

      ```
      public static void findgrains(org.openide.filesystems.FileObject[] fileObjects)
      ```

      Detect the grains of a set images.

      Parameters:
      :   `fileObjects` - and array of image files

      See Also:
      :   `FileObject`


- Package
- Class
- Tree
- Deprecated
- Index
- Help

*org.basmati.workspace 1.0*

- Prev Class
- Next Class

- Frames
- No Frames

- All Classes

- Summary:
- Nested |
- Field |
- Constr |
- Method

- Detail:
- Field |
- Constr |
- Method
